# Prognostic analysis of breast cancer in Xinjiang based on Cox proportional hazards model and two−step cluster method

**DOI:** 10.3389/fonc.2022.1044945

**Published:** 2023-01-17

**Authors:** Mengjuan Wu, Ting Zhao, Qian Zhang, Tao Zhang, Lei Wang, Gang Sun

**Affiliations:** ^1^ Country College of Public Health, Xinjiang Medical University, Urumqi, China; ^2^ Department of Medical Record Management, The Affiliated Cancer Hospital of Xinjiang Medical University, Urumqi, Xinjiang, China; ^3^ Information Management and Big Date Center, The Affiliated Cancer Hospital of Xinjiang Medical University, Urumqi, Xinjiang, China; ^4^ Department of Medical Engineering and Technology, Xinjiang Medical University, Urumqi, China; ^5^ Xinjiang Cancer Center/Key Laboratory of Oncology of Xinjiang Uyghur Autonomous Region, Urumqi, Xinjiang, China; ^6^ Department of Breast and Thyroid Surgery, The Affiliated Cancer Hospital of Xinjiang Medical University, Urumqi, Xinjiang, China

**Keywords:** breast cancer, prognostic model, survival analysis, nomogram, two-step cluster analysis

## Abstract

**Objective:**

To examine the factors that affect the prognosis and survival of breast cancer patients who were diagnosed at the Affiliated Cancer Hospital of Xinjiang Medical University between 2015 and 2021, forecast the overall survival (OS), and assess the clinicopathological traits and risk level of prognosis of patients in various subgroups.

**Method:**

First, nomogram model was constructed using the Cox proportional hazards models to identify the independent prognostic factors of breast cancer patients. In order to assess the discrimination, calibration, and clinical utility of the model, additional tools such as the receiver operating characteristic (ROC) curve, calibration curve, and clinical decision curve analysis (DCA) were used. Finally, using two-step cluster analysis (TCA), the patients were grouped in accordance with the independent prognostic factors. Kaplan-Meier survival analysis was employed to compare prognostic risk among various subgroups.

**Result:**

T-stage, N-stage, M-stage, molecular subtyping, type of operation, and involvement in postoperative chemotherapy were identified as the independent prognostic factors. The nomogram was subsequently constructed and confirmed. The area under the ROC curve used to predict 1-, 3-, 5- and 7-year OS were 0.848, 0.820, 0.813, and 0.791 in the training group and 0.970, 0.898, 0.863, and 0.798 in the validation group, respectively. The calibration curves of both groups were relatively near to the 45° reference line. And the DCA curve further demonstrated that the nomogram has a higher clinical utility. Furthermore, using the TCA, the patients were divided into two subgroups. Additionally, the two groups’ survival curves were substantially different. In particular, in the group with the worse prognosis (the majority of patients did not undergo surgical therapy or postoperative chemotherapy treatment), the T-, N-, and M-stage were more prevalent in the advanced, and the total points were likewise distributed in the high score side.

**Conclusion:**

For the survival and prognosis of breast cancer patients in Xinjiang, the nomogram constructed in this paper has a good prediction value, and the clustering results further demonstrated that the selected factors were important. This conclusion can give a scientific basis for tailored treatment and is conducive to the formulation of focused treatment regimens for patients in practical practice.

## 1 Introduction

Breast cancer is one of the most prevalent malignant tumors, which is the major death cause of cancer worldwide (1). Nearly 2,3 million women were diagnosed with breast cancer, representing 11.7% of all new cases, as reported by the World Cancer Statistics in 2020 (1). It is estimated that the number of new breast cancer patients in China in 2022 will reach 429,105, of which the number of death cases is 124,002 (2). In Xinjiang, the frequency of breast cancer has increased in recent years (3), posing a grave threat to the health and safety of women.

Breast cancer has highly heterogeneous pathological features. Tumors of some breast cancer patients develop slowly and have a good prognosis, whereas others have aggressive tumors with a bad prognosis (4). The clinical prognosis of breast cancer is primarily predicted by conventional clinical assessment methods based on TNM staging, claims the 8th edition of the Cancer Staging Manual issued by the American Joint Committee for Cancer (5). It has been established that some parameters, including the patient’s age, clinical features, tumor classification based on molecular analysis, distant metastatic site, and treatment, should be considered when determining the prognosis of breast cancer (6–8). Therefore, it is necessary to establish an effective model to forecast the prognosis of breast cancer by incorporating multiple parameters.

Machine learning (9), Mendelian randomization (10), nomograms (11, 12), etc., are often used techniques for tumor prediction. Nomograms, in particular, can substitute complicated regression formulas with straightforward, simple-to-understand visual graphs, making it possible to quickly determine the likelihood of a certain outcome event (such as a patient’s death or cancer recurrence) (13, 14). Numerous research studies employing nomograms to investigate the prognosis of breast cancer have been conducted (15–19). For instance, Fan et al. (15) investigated the clinical traits of HER2-positive breast cancer patients, and used a nomogram to correctly forecast the patients’ survival for their particular type of breast cancer. To differentiate and forecast whether breast cancer patients had liver metastases, a nomogram was created in (16). Su et al. (18) based on the differentially expressed genes between normal breast tissue and cancer tissue, developed a 19-genes-based prognostic signature and nomogram of patient survival prognosis. However, the majority of these studies used data from SEER, TCGA, and other public databases, which had fewer clinical Asian racial data (20, 21). There might be some restrictions when directly implementing established models. Studies on the clinicopathological traits and prognostic variables of female breast cancer patients in various parts of China have also been conducted in recent years (22–24). However, there aren’t many prognostic studies on breast cancer in Xinjiang. Therefore, analyzing the clinical information of Xinjiang patients with breast cancer to examine prognostic variables is important.

The method that has been widely used in cancer clustering, the cluster analysis, can be used to study the epidemiological characteristics of cancers, the different types of cancer, and the symptom clusters of patients (25–27). In order to make objects within a class as homogeneous as feasible and those between classes as heterogeneous as possible, the data is divided into various classes in accordance with predetermined criteria (28). For instance, Montazeri et al. (25) employed spatial cluster analysis to examine the patterns of occurrence of potentially high-risk breast and prostate cancer clusters in southern Iran. The data of patients with triple-negative breast cancer were reclassified by Liu et al. using spectral, hierarchical agglomerative, and K-means clustering, respectively (27). This gave the associated research on the prognosis of breast cancer a new direction.

As is common knowledge, there are variations in the incidence and mortality of cancer according to geographic regions. Clinical traits, prognostic variables, and survival rates of breast cancer patients in Xinjiang are unique from those in other regions due to lifestyle and culture (3, 29, 30). Related studies (31–33) have revealed that the incidence of breast cancer in Xinjiang is marginally lower than that of the entire nation, with the features that Luminal B breast cancer is more prevalent, the proportion of HER-2 overexpression type is lower, and triple-negative breast cancer is more likely to relapse and metastasize in Uygur patients. Consequently, it is crucial to concentrate on the prognosis of breast cancer patients in Xinjiang.

Motivated by the aforementioned research, the prognostic markers for breast cancer patients were identified in this paper utilizing information from patients who were diagnosed with the disease at the Affiliated Cancer Hospital of Xinjiang Medical University between 2015 and 2021. Additionally, a nomogram of the 1-, 3-, 5-, and 7-year overall survival rates of breast cancer patients was created. Another concern issue in our paper is to cluster all breast cancer patients and compare the dissimilarity between various clusters. One of the most crucial clustering techniques is two-step cluster analysis (TCA), which tries to identify natural groups (or clusters) in the data set that are not immediately apparent. TCA, on the other hand, can analyze combinations of several types of variables (34). As a result, in our study, TCA is applied to cluster the patients based on independent prognostic markers that are discovered using nomogram analysis, and then to assess the clinicopathological characteristics and prognosis risk in various subgroups. Our findings offer a precise assessment of the probability of survival for breast cancer patients in Xinjiang and give medical professionals a solid foundation on which to build specialized treatment regimens for each patient.

## 2 Objects and methods

### 2.1 Research objects

#### 2.1.1 Patient information extraction

Data of 8226 patients diagnosed with breast cancer for this study is sourced from the Affiliated Cancer Hospital of Xinjiang Medical University, which is the only tumor special center in Xinjiang. The patients in this hospital come from all over Xinjiang and thus the data used in this study is representative to a certain extent. They were followed up by contacting, messaging, and confirming the information for outpatient and inpatient patients. 96.62% of follow-ups were actually effective. The seven-year data ranges from January 1, 2015, to December 31, 2021.

Basic demographic, clinicopathological and survival information on patients were gathered, including the age at diagnosis, marital status, length of hospital stays, histological grade, molecular subtypes, clinical stage, T-stage, N-stage, M-stage, as well as receiving targeted therapy, postoperative chemotherapy, postoperative radiotherapy, postoperative targeting, axillary lymph node dissection, the type of operation. **Supplementary Table 1** displays the variable names and assignments for the factors. T-stage (primary tumor) was split into T1(tumor size ≤ 2 cm), T2(>2 to 5 cm), T3(>5 cm), and T4 disease was defined as a tumor of any size with direct extension to the chest wall and/or to the skin (ulceration or skin nodules, macroscopic nodules); N-stage (regional lymph nodes) was divided into N0, N1, N2, and N3 (classification standard: N0, no regional lymph node metastases; N1, micrometastases; or metastases in 1-3 axillary lymph nodes; N2, metastases in 4-9 axillary lymph nodes; N3, metastases in 10 or more axillary lymph nodes); and M-stage (distant organ metastases) was divided into M0(no clinical or radiographic evidence of distant metastases), M1(distant metastases). These divisions were made in accordance with the 8th edition of the American Joint Committee on Cancer standards (35). The time period from the date of diagnosis to the date of patient death or the study cutoff was the primary endpoint of this trial, which was defined as overall survival.

#### 2.1.2 Patients selection

**Figure 1:**
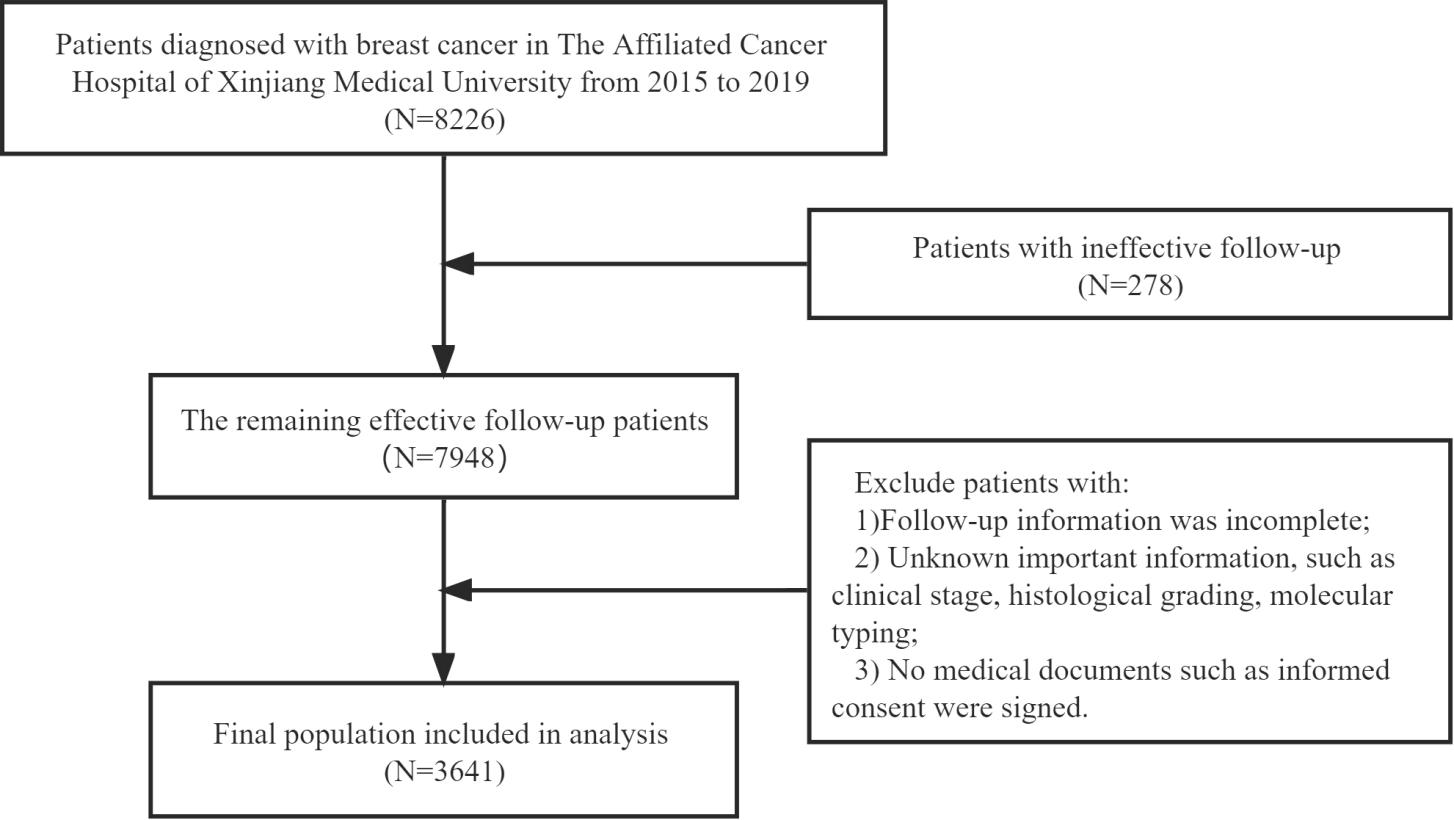
Flow chart of patient selection.

Patients were included if they met all three criteria: 1) were above the age of 18; 2) had only primary site tumor that was pathologically identified as BC; and 3) had complete clinicopathological and follow-up information. The following were the exclusion criteria: 1) patients with inadequate follow-up information, 2) unsigned medical documents, such as informed consent and patient instructions, at the time of admission; and 3) the absence of critical information, such as molecular subtypes, clinical stage, and surgical treatment approach. The instances fulfilling the criteria were gradually screened out in accordance with the inclusion and exclusion criteria (see **Figure 1**).

In the total of 8226 patients, the number of ineffective follow-up patients is 278 (for some uncontrollable reason, the patient or family members cannot be contacted. This kind of data cannot be used to count the outcome and calculate the survival rate). According to the inclusion and exclusion criteria, 4307 patients were excluded. For instance, there were 1218 individuals missing molecular typing information, and 2180 individuals with the lack of histological grade information. Therefore, there were 3641 patients included in the final analysis.

Riley et al. in 2018 (36) proposed criteria were used to determine the minimal sample size needed for the construction of clinical prediction models. The study used 17 different independent variables in total. A follow-up of 4.47 years on average was expected, with a follow-up length of 7 years anticipated. Incidence of the outcome was 0.089, and estimated R-squared was 0.08. Using these numbers as a guide, we determined that 1827 samples, corresponding to 8167 follow-ups, would be the bare minimum needed to create the new model. An adequate sample size of 3641 patients was gathered for this study.

### 2.2 Statistical analysis

#### 2.2.1 Difference analysis

Using the R language’s random sample function, eligible patients were arbitrarily split into training and validation groups in a 7:3 ratio. Between the two groups, comparisons and analyses of each variable’s baseline characteristics were conducted. When the quantitative data fit the normal distribution, the mean and standard deviation (SD) were employed, and the t-test was performed to examine the difference between groups. The Wilcoxon rank-sum test was used to compare non-normally distributed data that were reported as the median (first quartile, third quartile). The chi-square test was used to compare categorical data that were expressed as frequency (constituent ratio). SPSS (IBM SPSS Statistics) was used to carry out the analyses. In this investigation, a two-sided p-value of 0.05 or lower was regarded as statistically significant.

#### 2.2.2 Cox proportional hazard model

The most often used survival regression model for examining the connection between predictors and time-to-event is the Cox proportional hazards model (37). The basic form of the Cox regression model:


$$h\left(t,X\right)=h_{0}\left(t\right)exp\left(\beta_{1}X_{1}+\beta_{2}X_{2}+\cdots +\beta_{n}X_{n}\right),$$

Calculate the Hazard ratios (HR) according to the parameter *β*, *HR=exp*(*β*). *HR* were applied to identify protective factors (*HR<1*) and risk factors (*HR>1*), a *HR* of 1 means the factor has no effect on the hazard of the event.

With the Cox proportional hazard regression model, both univariate and multivariate proportional analyses were run. Statistically significant factors (P< 0.05) in univariate analysis and factors with clinical practice value were added to the multivariate Cox regression model on the basis of this initial screening. Finally, it was possible to identify the independent risk factors influencing the survival and prognosis of patients in the training group.

#### 2.2.3 Development and validation of nomogram

A nomogram is a convenient graphical representation of a mathematical model, in which various important factors are combined to forecast the overall survival. Nomograms have the ability to build a practical bridge between clinicians and patients. We can apply the clinical nomograms to clinical practice quickly and concisely according to the patient’s specific characteristics. The nomogram of 1-, 3-, 5- and 7-year survival probability of patients in the training group was constructed based on the findings of the Cox regression analysis. In the training and validation groups, the model was assessed in accordance with three different criteria. First, the Receiver operating characteristic (ROC) curve area under curve (AUC), which has values between 0.5 and 1.0, was used to assess the nomogram’s discrimination. And the discrimination is better the higher the value of AUC. Second, the calibration curve was used to gauge how well the model was calibrated. The model’s projected value was more closely aligned with the actual observed value the closer the curve was to the 45° reference line, which indicated a higher degree of nomogram calibration performance. Finally, the decision curve analysis (DCA) was used to assess the clinical utility of the nomogram based on threshold probability (14). In the meantime, using the nomogram, it is possible to determine the total point for each patient, that is, the total point of the corresponding individual scores after taking into account the values of all variables. The likelihood that a patient will survive is inversely correlated with the total point. Using R language software (version 4.1.3), the nomogram, ROC curves, calibration curves, and DCA were created.

#### 2.2.4 Two-Step cluster analysis

The precise calculation procedures for the Two-Step cluster analysis in SPSS are as follows (38, 39). Establishing the clustering feature tree is the first step (pre-clustering). The values for the first variable in the data set are shown on a leaf node of the tree root, and the similarity criteria is the distance measurement. Recursive induction is used to create the clustering feature tree after merging the new node in accordance with how similar the variable and the old node are. The distance measurement model adopts log-likelihood, and the calculation formula is


$$d\left(i,j\right)=\zeta_{i}+\zeta_{j}-\zeta_{i,j}$$

where *d*(*i*,*j*) is the distance between two clusters *i* and *j*, *ζ_i_* and *ζ_j_* are the likelihood function values of cluster *i* and *j* respectively. *i*,*j* is the new cluster generated by the combination of clusters *i* and *j*.


$$\zeta_{v}=-N_{V}\left(\sum_{k=1}^{K^{A}}\frac{1}{2}\log\left({\hat{\sigma }}_{k}^{2}+{\hat{\sigma }}_{vk}^{2}\right)+\sum_{k=1}^{K^{B}}{\hat{E}}_{vk}\right)$$

where *N_v_* is the number of data records in cluster *v*. *K^A^* is the total number of all continuous variables; *K^B^* is the total number of all categorical variables; ${\hat{\sigma }}_{k}^{2}$ is the estimated variance of the *k*th continuous variable in the whole data set; ${\hat{\sigma }}_{vk}^{2}$ is the estimated variance of the *k*th continuous variable in cluster *v*; ${\hat{\text{E}}}_{vk}$ is the estimated mean of the *k*th continuous variable in cluster *v*.


$${\hat{E}}_{vk}=-\sum_{l=1}^{L_{k}}\frac{N_{\upsilon kl}}{N_{\upsilon }}\log\frac{N_{\upsilon kl}}{N_{\upsilon }}$$

where *L_k_* represents the number of categories of the *k*th categorical variable; *N_vkl_* is the number of data records in *l* category of the *k*th categorical variable in cluster *v*.

The combined clustering algorithm is used to combine the leaf nodes in the second stage (clustering), which might result in a collection of clustering schemes with various numbers of clusters. Automatically select the clustering scheme with the best clustering number in accordance with the BIC (Bayesian information criterion) rules for a range of clustering scheme comparisons. For cluster *J*, calculating formula for BIC


$$BIC\left(J\right)=-2\sum_{j=1}^{J}\zeta_{j}+m_{J}\log\left(N\right)$$

$$m_{J}=J\left\{2K^{A}+\sum_{k=1}^{K^{A}}\left(L_{k}-1\right)\right\}$$

where *N* representative data set on the number of records; $\sum_{j=1}^{J}\zeta_{j}$ is the maximum likelihood function; *m_J_* is the number for the model parameters.

## 3 Results

### 3.1 Difference analysis results

This study had 3641 patients in total, with a training group (2540) and a validation group (1101). The median follow-up time was 1620 days for the training group and 1650 days for the validation group, respectively. The follow-up duration for each group was 0~2920 days and 16~2862 days. **Supplementary Table 2** displays the clinicopathological traits of the patients in the training and validation group, in which it can be found that the proportion of patients with histological grade two, Luminal B, lower T-, N-, and M-stage, and patients who choose radical surgery are more.

The quantitative data in the sample all followed a normal distribution, and the baseline characteristics of the two groups were compared using the chi-square and t tests. Except for the tumor’s histological grade, the results showed that there was no difference between the two groups in the other factors (P>0.05), indicating that the two groups were comparable.

### 3.2 Results of Cox regression analysis

The survival prognosis of 2,540 patients in the training group was related to tumor histological grade, molecular subtype, clinical diagnosis stage, T-stage, N-stage, M-stage, tumor metastasis, postoperative chemotherapy, postoperative radiotherapy, and operation type (P<0.05) according to univariate Cox regression analysis. It was unrelated to the other variables (P>0.05). Six independent prognostic markers, including molecular subtypes, T-stage, N-stage, M-stage, postoperative chemotherapy, and operation type, were identified by performing multivariate Cox regression analysis on the basis of the results of univariate analysis and clinical considerations (**Table 1**).

**Table 1:** Univariate and multivariate Cox regression analysis in breast cancer patients.

Characteristic	Univariate			Multivariate		
	HR	Z	P	HR	95% CI	P
**Age**	1.008981	1.494	0.135			
**Marital status**						
unmarried	—	—				
married	1.005814	0.02	0.984			
**Length of hospital stay**	1.003699	0.311	0.756			
**Histology grade**						
Grade1	—	—				
Grade2	1.9227	1.439	0.1503			
Grade3	2.6923	2.146	<0.05			
**Molecular subtyping**						
Luminal A	—	—		—	—	
Luminal B	1.7438	2.037	<0.05	1.32	0.77, 2.28	0.3
HER2 overexpressing	3.3421	4.011	<0.05	2.03	1.10, 3.77	<0.05
Triple negative	2.9775	3.738	<0.05	2.43	1.36, 4.36	<0.05
**Clinical stage**						
I	—	—				
II	2.4585	3.721	<0.05			
III	7.7218	8.655	<0.05			
IV	34.5028	13.424	<0.05			
**T-stage**						
T1	—	—		—	—	
T2	2.9424	6.306	<0.05	1.87	1.32, 2.66	<0.05
T3	10.804	10.977	<0.05	4.6	2.89, 7.32	<0.05
T4	11.2956	10.023	<0.05	3.45	2.06, 5.78	<0.05
N-stage						
N0	—	—		—	—	
N1	3.1156	6.106	<0.05	2.55	1.75, 3.72	<0.05
N2	4.9914	7.582	<0.05	3.77	2.44, 5.83	<0.05
N3	10.7176	12.139	<0.05	5.39	3.51, 8.27	<0.05
**M-stage**						
M0	—	—		—	—	
M1	10.3376	14.06	<0.05	3.23	2.22, 4.71	<0.05
**Tumor recurrence**						
No	—	—				
Yes	1.8419	0.609	0.542			
**Tumor metastasis**						
No	—	—				
Yes	2.7566	7.535	<0.05			
**Receiving targeted therapy**						
No	—	—				
Yes	1.1837	1.018	0.309			
**Operation type**						
No	—	—		—	—	
Breast conserving surgery	0.1232	7.396	<0.05	0.35	0.20, 0.63	<0.05
Radical operation	0.2964	7.905	<0.05	0.46	0.33, 0.64	<0.05
**Axillary lymph node dissection**						
No	—	—				
Yes	1.1224	0.842	0.4			
**Postoperative chemotherapy**						
No	—	—		—	—	
Yes	0.6816	2.423	<0.05	0.45	0.32, 0.62	<0.05
**Postoperative radiotherapy**						
No	—	—				
Yes	0.7248	2.302	<0.05			
**Postoperative targeting**						
No	—	—				
Yes	0.7612	1.266	0.205			

The research results showed that compared with Luminal A type patients, the risk of death from cancer in Luminal B type (HR=1.32, 95% CI: 0.77 to 2.28), HER2 overexpressing (HR=2.03, 95% CI: 1.10 to 3.77), and Triple negative (HR=2.43, 95% CI: 1.36 to 4.36) patients were higher. The T-stage (T2, HR =1.87, 95% CI: 1.32 to 2.66; T3, HR =4.6, 95% CI: 2.89 to 7.32; T4, HR =3.45, 95% CI: 2.06 to 5.78), N-stage (N1, HR =2.50, 95% CI: 1.75 to 3.72; N2, HR =3.77, 95% CI: 2.44 to 5.83; N3, HR =5.39, 95% CI: 3.51 to 8.27), M-stage (M1, HR =3.23, 95% CI: 2.22 to 4.71) were associated with the mortality of patients with breast cancer. Patients who underwent breast conserving surgery (HR=0.35, 95%CI: 0.20 to 0.63) and radical surgery (HR=0.46, 95%CI: 0.33 to 0.64) had a lower risk of death than those who did not undergo surgery. Patients with participation in postoperative chemotherapy had a lower risk of death than in patients without (HR=0.45, 95% CI: 0.32, 0.62), which was a protective factor for patients.

### 3.3 Construction and verification of a prognostic nomogram

**Figure 2:**
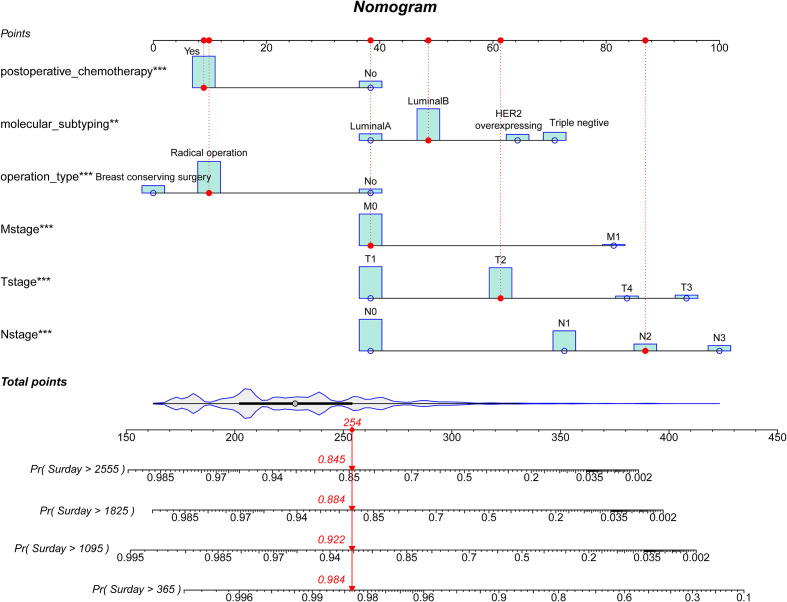
A prognostic nomogram for breast cancer patients. HER2, Human epidermal growth factor receptor 2. A patient will be randomly selected here, the red dashed line corresponds to the specific clinical characteristics of the patient, and the individual score of the variable projected to the Points in the first row; The total score of all indicators is “Total Point”; Finally, the survival probability of patients after 1-, 3-, 5- and 7-year can be obtained according to the total score.

A nomogram was created using the variables with statistically significant data from the multivariate Cox regression analysis to forecast the 1-, 3-, 5- and 7-year survival rates of breast cancer patients (**Figure 2**). The red dashed line depicted the likelihood that a randomly selected patient in the sample would survive, and the nomogram model demonstrated that N-stage had the biggest influence on the prognosis of breast cancer in Xinjiang.

**Figure 3:**
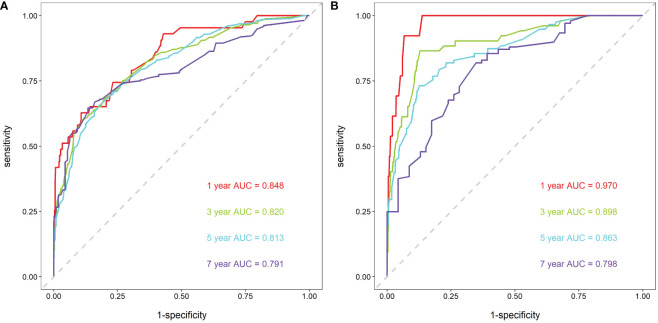
ROC curves for breast cancer patients. **(A)** ROC curves of 1-, 3-, 5- and 7-year in the training group, **(B)** ROC curves of 1-, 3-, 5- and 7-year in the validation group.

**Figure 4:**
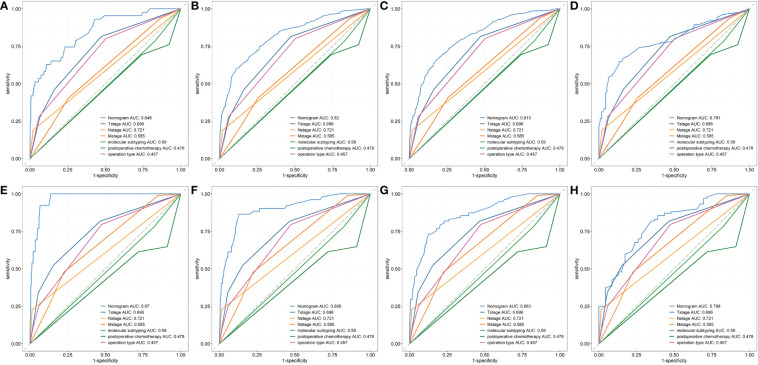
The ROC curves of nomogram and all independent predictors at 1- **(A)**, 3- **(B)**, 5-**(C)** and 7-year **(D)** in the training group and at 1- **(E)**, 3- **(F)**, 5-**(G)** and 7-year **(H)** in the validation group.

**Figure 5:**
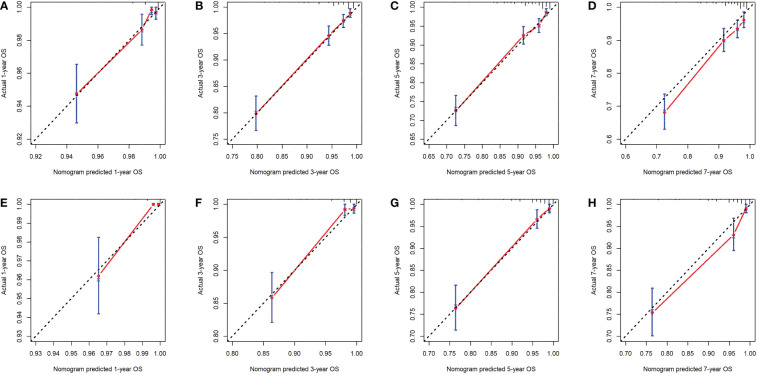
The calibration curves of the nomogram for the 1-, 3-, 5- and 7-year OS prediction of the training group **(A-D)** and validation group **(E-H)**.

**Figure 6:**
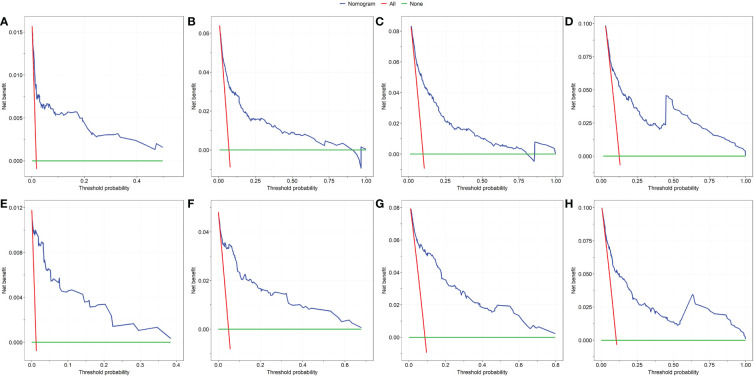
The DCA of the nomogram for the survival prediction of breast cancer patients. 1-year **(A)**, 3-year **(B)**, 5-year **(C)** and 7-year **(D)** survival benefit in the training group. 1-year **(E)**, 3-year **(F)**, 5-year **(G)** and 7-year **(H)** survival benefit in the validation group.

First, ROC curves of the training and validation group’s 1-, 3-, 5- and 7-year overall survival rates were created in order to validate the nomogram. The prognostic model’s AUC values were respectively 0.848, 0.820, 0.813, and 0.791 in the training group and 0.970, 0.898, 0.863, and 0.798 in the validation group (**Figure 3**). The nomogram was also compared to all independent prognostic factors in the two groups, and ROC curves were plotted as a result. The results showed that the nomogram’s AUC values were higher than those of all independent prognostic factors in the 1-, 3-, 5- and 7-year time periods, as shown in **Figure 4**. The calibration curve was also produced simultaneously to assess the degree of calibration of the nomogram in the two groups. In the training and validation group, the calibration curves for the first, third, and fifth years of overall survival were relatively near to the ideal 45° reference line (**Figure 5**). The findings show that the nomogram has a good calibration function and that the model’s predicted values and actual values correspond well. Last but not least, DCA curves were drawn based on the survival and prognosis of the two groups (**Figure 6**). The blue curve, which calculates the net benefit of the nomogram model by adding the true positives and deducting the false positives, while the red curve represents the assumption that all patients will die (12). The findings show that the nomogram prediction model has significant clinical utility.

### 3.4 Results of Two-Step cluster analysis

The following were the important TCA parameters using SPSS: Molecular subtype, T-stage, N-stage, M-stage, involvement in postoperative care, and type of surgery were categorical variables. Total Point, a new continuous variable, was obtained using the sum of all patients’ scores as determined by the nomogram. The outcomes are displayed in **Supplementary Figure 1**. Two clustering groups are the ideal number, and the clustering quality is Fair.

**Figure 7:**
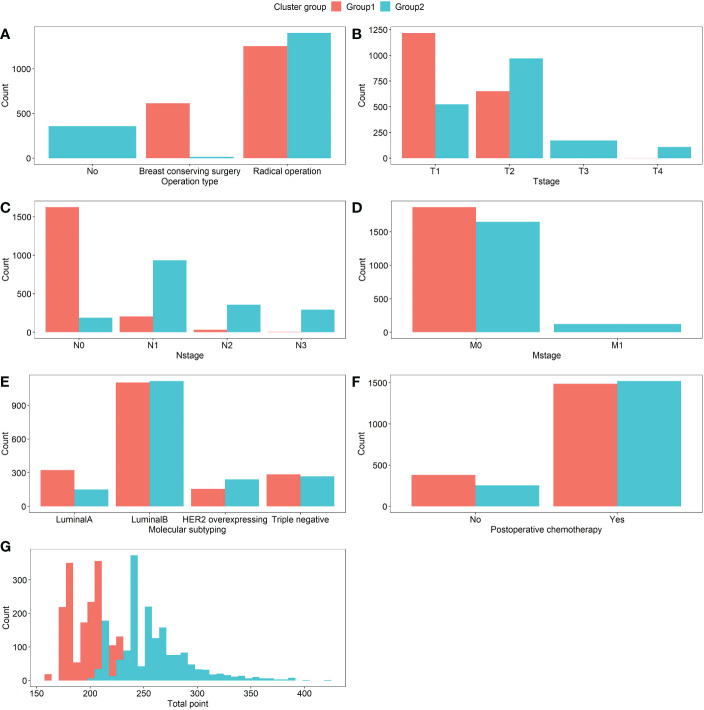
Comparison of the distribution of variables between Group1 and Group2 patients, which were **(A)** Operation type, **(B)** T-stage, **(C)** N-stage, **(D)** M-stage, **(E)** Molecular subtyping, **(F)** Postoperative chemotherapy, and **(G)** Total point, respectively. The count means the number of patients in each variable.

Group1 and Group2 were given to the groupings that resulted from clustering, respectively. As seen in **Figure 7**, Group1 demonstrated that breast conserving surgery and radical surgery was more prevalent (**Figure 7A**), that the T-, N-, and M-stages were concentrated in the early T1, T2, N0, N1, and M0 stages (**Figures 7B–D**), with a lower total point of nomogram (**Figure 7G**). The majority of the patients in Group2 did not receive surgical therapy (**Figures 7A**), the T-, N-, and M-stages were more advanced (**Figures 7B–D**), and the total point distribution was likewise on the high score side (**Figure 7G**). And there was no significant difference in the distribution of molecular typing and postoperative chemotherapy between the two groups (**Figures 7E, F**).

As indicated in **Table 2**, the reliability of the above classification was further confirmed and the difference between the two groups was statistically significant (P<0.05).

**Table 2:** Comparison of the distribution of variables between Group1 and Group2 after clustering.

	Group1	Group2	t/*χ* ^2^	P
	(N=1869)	(N=1772)		
**Total point**				
Mean ± SD	200.273 ± 20.246	258.467 ± 33.214	64.204	<0.001
**Molecular subtyping**				
Luminal A	323(17.3%)	150(8.5%)	78.817	<0.001
Luminal B	1103(59.0%)	1115(62.9%)		
HER2 overexpressing	157(8.4%)	240(13.5%)		
Triple negative	286(15.3%)	267(15.1%)		
**T-stage**				
T1	1217(65.1%)	523(29.5%)	614.113	<0.001
T2	651(34.8%)	969(54.7%)		
T3	0(0.0%)	171(9.7%)		
T4	1(0.1%)	109(6.2%)		
**N-stage**				
N0	1625(86.9%)	189(10.7%)	2146.618	<0.001
N1	206(11.0%)	934(52.7%)		
N2	32(1.7%)	357(20.1%)		
N3	6(0.3%)	292(1635%)		
**M-stage**				
M0	1869(100%)	1649(93.1%)	134.269	<0.001
M1	0(0.0%)	123(6.9%)		
**Postoperative chemotherapy**				
No	381(20.4%)	254(14.3%)	23.132	<0.001
Yes	1488(79.6%)	1518(85.7%)		
**Operation type**				
No	0(0.0%)	357(20.1%)	938.580	<0.001
Breast conserving surgery	611(33.0%)	14(0.8%)		
Radical operation	1253(67.0%)	1401(79.1%)		

### 3.5 Kaplan-Meier curve

**Figure 8:**
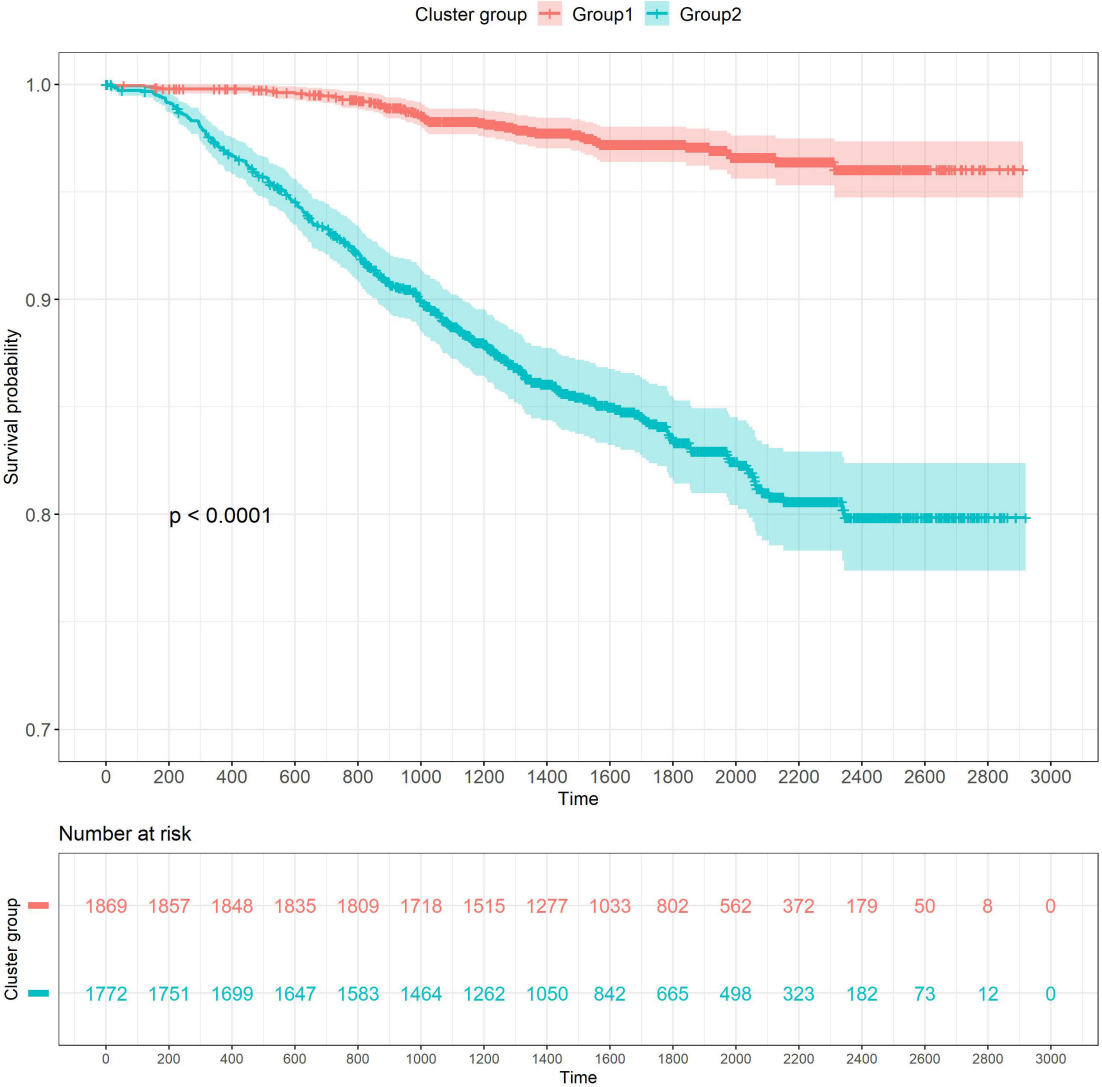
Kaplan–Meier survival analysis of the signature for both Group 1 and Group 2.

The results of the Kaplan-Meier survival analysis demonstrated that there were notable variations in the overall survival curves between various subgroups of breast cancer patients. There was a statistically significant difference in the prognosis between the two subgroups (Log-rank test, *χ* ^2^ = 186, P<0.001), and the curve of Group2 was lower than that of Group1 (**Figure 8**). It further indicated that patients with worse prognoses included those with higher total points, T-, N-, and M-stages in advanced disease, as well as those who did not receive operational treatment or postoperative chemotherapy, which was consistent with the findings of the Cox proportional hazards model.

Furthermore, the survival curves were constructed according to the different drugs and regimens used by patients in postoperative chemotherapy (**Supplementary Figures 2**, **3**). Chemotherapy regiments are divided into mono therapy and multi-drug combination therapy. The results demonstrated that compared with patients who did not receive chemotherapy, the survival rates of patients treated with mono and combination therapy were higher, especially for patients treated with mono therapy (and these drugs mainly include taxoids, anthracyclines and platinum compounds). The results showed that the survival rate of patients with taxoids treatment was higher than that of patients without taxoids treatment, while the survival rate of patients with platinum compounds treatment was the opposite. And the use of anthracyclines or not had no significant effect on patients’ survival.

## 4 Discussion

In Xinjiang, there is a high prevalence of breast cancer and an upward trend in incidence over time (3, 40). The clinical information of breast cancer patients in Xinjiang between 2015 and 2021 was analyzed, and the independent prognostic indicators of breast cancer were confirmed as T-stage, N-stage, M-stage, molecular subtyping, type of operation, and involvement in postoperative chemotherapy by using Cox regression analysis. The six predictive factors formed the nomogram, which was built. According to the verification results, this nomogram had good discrimination, calibration, and clinical application value in both the training and validation groups, indicating that the model could be used to predict the survival of breast cancer patients in Xinjiang. In addition, the TCA approach was used to split breast cancer patients into two subgroups (Group1 and Group2) by taking into account the six independent prognostic indicators and the total point of the nomogram. In particularly, the group with the worse prognosis, (in which the majority of patients did not undergo surgical therapy or postoperative chemotherapy), the T-, N-, and M-stage were more prevalent in the advanced, and the total points were likewise distributed in the high score side. The survival probability was assessed using Kaplan-Meier survival curves, which revealed that the overall survival rates of the two subgroups were substantially different (Log-rank test, P<0.001) and that the prognosis of Group2 was poorer than that of Group1. This further verified the dependability of the nomogram.

Another interesting result in this paper is that triple-negative breast cancer patients had the worst prognosis, and the survival rate was generally lower than that of patients with other types, which is consistent with the findings of (41). Triple negative breast cancer patients in Xinjiang have a characteristic with high recurrence and metastatic rates (32), so it is necessary to take more aggressive treatment for these patients. Additionally, it was discovered that the T-, N-, and M-stages are independent prognostic factors. The riskiest T-stage was T3(HR =4.6, 95% CI: 2.89 to 7.32), followed by T4(HR =3.45, 95% CI: 2.06 to 5.78), there was little difference in risk between the two subperiods. According to earlier research, bigger tumors have more tumor cells and have a worse prognosis (42, 43). Because the awareness of self-protection for patients has been enhanced through early cancer screening and health education, the lumps can be early detected when they are small, and there are few patients whose tumors grow as large as the T4 substage. The data in our paper showed that the sample sizes of patients with T3 and T4 are very small, only accounting for 4.7% and 3%, respectively. On the other hand, the actual size of tumor in T4 substage may be small and the location and growth direction of this tumor would be affected by its size. Thus, the prognosis of patients in T4 substage would not be much worse. The clinical stages of breast cancer patients in Xinjiang are concentrated in the early stages (3), which may be the cause of the results in this paper being marginally different from those. As a result, the survival probability of patients with advanced stage differs slightly. And it was discovered that patients with N1, N2, and N3 subperiods had a higher probability of dying from cancer than patients with N0 subperiods did. The more advanced the N-stage, the more lymph node metastasis and the number of metastases, and then the higher the risk of mortality, which is consistent with previous studies that lymph node metastasis is a frequent clinical characteristic of breast cancer progression (19, 44). Given that the HR for M1 in the M-stage was higher than 1 (HR=2.96, 95% CI: 2.04 to 4.29), indicating that distant metastasis is a risk factor for survival. Therefore, routine screening for those who have breast cancer is advocated in order to achieve early detection, early diagnosis, and early treatment, reduce the mortality of breast cancer, and improve the prognosis and survival rate of patients. In addition, it was demonstrated in our findings that, when compared to no surgery, both breast conserving surgery and radical operation could greatly enhance the prognosis of patients, and the prognosis of breast conserving surgery is better than that of radical operation. Based on National Comprehensive Cancer Network guideline for treatment of breast cancer (45), patients would usually receive radiotherapy after breast conserving surgery, which can change the immune microenvironment of the tumor for patient. It is beneficial to improve the prognosis of the patient. Some research results (46, 47) show that for patients with early breast cancer, conservative surgery and radiotherapy provide the same survival period as radical surgery. On the other hand, because the stage of disease for patients who can perform breast conserving surgery is earlier than that of patients undergoing radical surgery, the prognosis for the former would be relatively good. Moreover, breast-conserving surgery is more advantageous for breast cancers due to lesser bleeding, rapid recovery, and fewer complications (48). Compared with modified radical surgery, the patients with breast conserving surgery can achieve better postoperative quality of life and psychological tolerance. However, breast-conserving surgery is not suitable for all breast cancer patients, patients need to choose the appropriate surgical method according to their actual condition and doctor’s advice. Our data showed that women are more likely to choose radical operation, which may be related to patients’ worries about postoperative disease recurrence and survival. From the patients’ point of view, however, breast conserving surgery can rank highly in maintaining quality of life. The findings of this study also showed that individuals with breast cancer who did not receive postoperative treatment had a much higher mortality risk. And it was proven that radiotherapy and chemotherapy were linked to the overall survival of people with breast cancer (17, 49). Surgical treatment of breast cancer can reduce the volume of the tumor and control the spread of the disease, effectively. When patients did not choose to receive surgical treatment, they can also choose chemotherapy treatment. However, chemotherapy couldn’t guarantee the therapeutic effect of breast cancer, and conservative treatment could only control the development of the disease to a certain extent. Therefore, radiotherapy or chemotherapy alone is not enough and the combination of surgery is needed. There are some possible reasons for the patients did not receive surgery, at first, it is possible that some patients have minimal breast disease. Secondly, some patients with advanced breast cancer might miss the best opportunity of surgical operation and could not receive the operation. And the others with poor general condition, severe organ disease or weakness are also not suitable for surgical treatment. In light of this, it is advised that patients seek surgical therapy as soon as possible after receiving a diagnosis. Patients can decide to have breast-conserving surgery based on the advice of their doctors and the clinical features of their disease. Some helpful measures of postoperative therapy after surgery could also improve the survival rate of patients.

Moreover, we analyzed the different drugs and regimens used by patients in postoperative chemotherapy. There are several chemotherapy drugs and regimens for breast cancer patients in clinical (50). During the treatment of the chemotherapy, patients usually choose a combination of multiple drugs, so it is unclear that what kind of medicine played a role. In this paper, the information collected on chemotherapy drugs is not complete enough, such as the information what types of drugs should be used for specific type of patients. Therefore, our results have a certain bias from the aspect of clinical treatment. This is also the limitations of this paper.

In (51), the whole cohort was randomly divided into a training cohort (n=113,996) and a testing cohort (n=113,993), with a ratio of about 1:1, so that the validation subcohort has the same origin as the training cohort. This method is a good way to get the better model results observed in the validation cohort than in the training cohort. In our future work, we will refer to this literature and collect more data (to reach the standard of large cohort) in order to make more effective prognostic analysis of breast cancer patients.

Additionally, the clinical data of breast cancer patients were only applied to analyze for the aim of prognosis prediction in this paper. However, by combining the imaging, survival and longitudinal data of breast cancer patients, some joint models for longitudinal and time-to-event data would be established to analyze the dynamic pattern of disease progression and more accurately predict the occurrence of events. On the other hand, we could extend the estimation framework of joint models in the context of case-cohort designs and create fresh approaches to effectively evaluate the prediction accuracy.

## 5 Conclusion

In conclusion, a prognostic nomogram was established in this paper based on the clinical information of breast cancer in Xinjiang, which could predict the 1-, 3-, 5- and 7-year overall survival rates of patients. The clinicopathological features and prognosis risk were evaluated by two-step cluster analysis in various subgroups. Our findings might potentially offer a solid scientific foundation for optimizing each patient’s individual therapeutic regimen.

## Data availability statement

The datasets presented in this article are not readily available because it can’t be used to publish a relevant article. Requests to access the datasets should be directed to TZhao, zhaoting0557@163.com.

## Author contributions

Conceptualization, MW and LW; Acquisition of data, TiZ, QZ, and GS; Methodology, LW and TaZ; Software, LW, MW, and TaZ; Writing-original draft, TiZ and MW; Writing-review & editing, MW, LW, GS, and QZ. All authors contributed to the article and approved the submitted version.
