# Supplementary material for: Prognostic analysis of breast cancer in Xinjiang based on Cox proportional hazards model and two−step cluster method
Source: Front Oncol. 2023 Jan 17;12:1044945. doi: 10.3389/fonc.2022.1044945 (PMC9887128; doi:10.3389/fonc.2022.1044945)
Supplement: Supplementary file 1 [file DataSheet_1.pdf]

Supplementary Material

Prognostic analysis of breast cancer in Xinjiang based on Cox proportional hazards model and two-step cluster method

1 Supplementary Information

Molecular classification of breast cancer:

According to the detection results of ER, PR, HER2 and Ki67 index, breast cancer was classified into four molecular subtypes as follows:

(1) Luminal A: ER positive, PR positive and high expression (>20%), HER2 negative and Ki-67 low expression (<14%);

(2) Luminal B (HER2 negative): ER positive and/or PR positive, HER2 negative, Ki-67 high expression (≥14%) or PR low expression (≤20%); Luminal B (HER2 positive): ER positive and/or PR positive, and HER2 positive (protein overexpression or gene amplification), Ki-67 in any state;

(3) HER2 overexpression: ER negative, PR negative and HER2 positive (protein overexpression or gene amplification);

(4) Triple-negative/Basal-like: Non-specific invasive ductal carcinoma, negative for ER, PR and HER2.

2 Supplementary Figures and Tables

2.1 Supplementary Figures

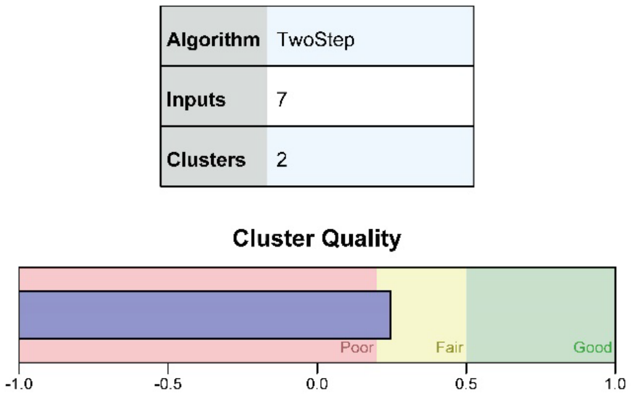

Supplementary Figure 1 Model summary and cluster quality.

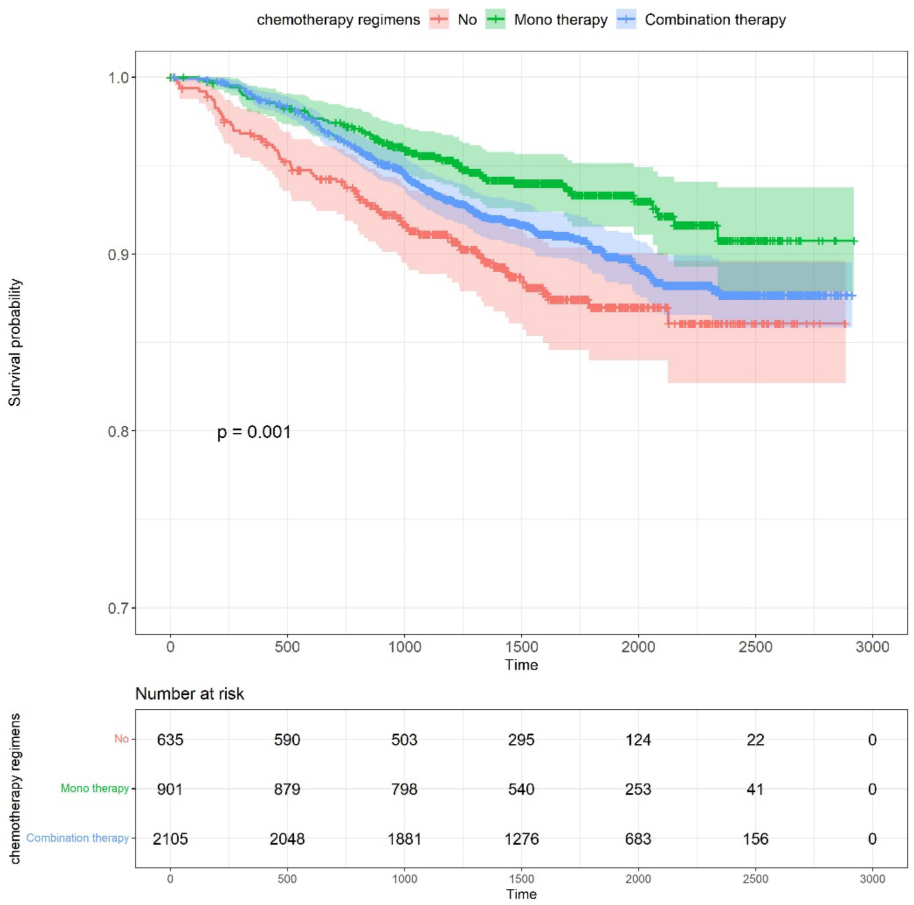

Supplementary Figure 2 Survival curve of patients with different chemotherapy regimens.

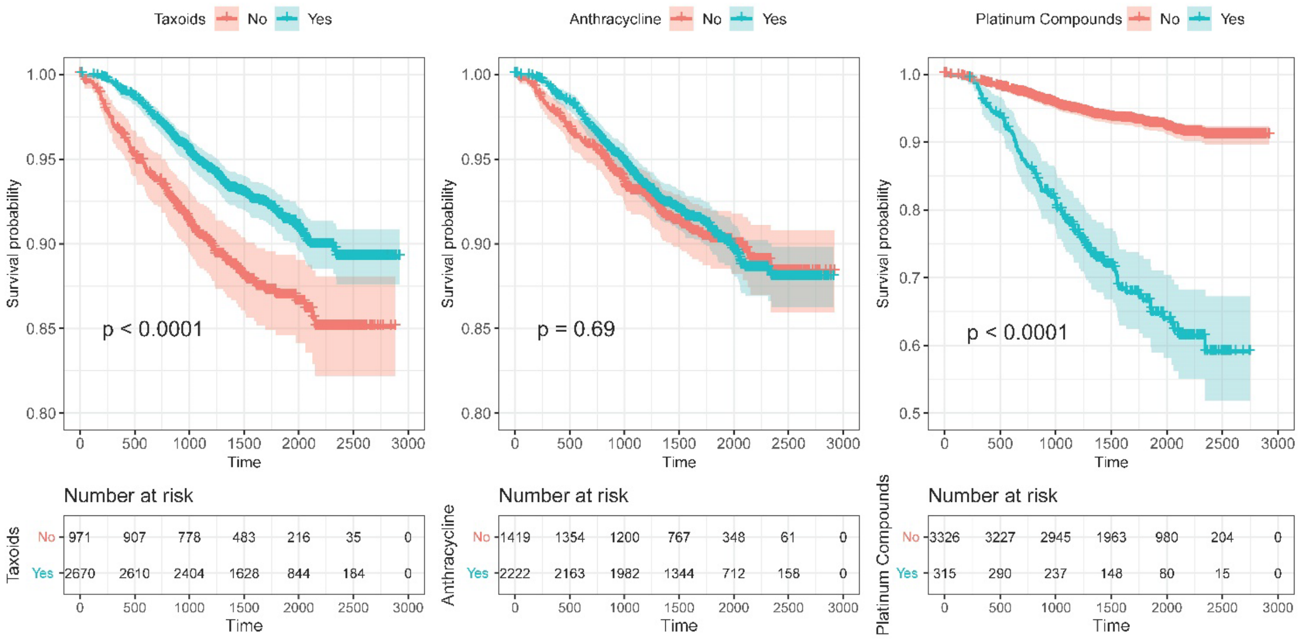

Supplementary Figure 3 Survival curve of patients with different chemotherapy drugs.

2.2 Supplementary Tables

Supplementary Table 1 Variable assignment table.

No.	Variable	Factor	Grouping and assignment
1	Surday	Survival time	day
2	death	Survival outcome	0=survival，1=death
3	age	Age	year
4	marital_status	Marital status	0=unmarried，1=married
5	in_hospital	Length of hospital stay	day
6	histology	Histological grade	1=Grade1，2=Grade2，3=Grade3
7	molecular_subtyping	Molecular subtype	1=Luminal A，2=Luminal B，3=HER2 overexpressing，4=Triple negative
8	TNM	Clinical stage	1=I，2=II，3=III，4=IV
9	Tstage	T-stage (Primary tumor)	1=T1，2=T2，3=T3，4=T4
10	Nstage	N-stage (Regional lymph nodes)	0=N0，1=N1，2=N2，3=N3
11	Mstage	M-stage (Distant organ metastases)	0=M0，1=M1
12	recur	Tumor recurrence	0=No，1=Yes
13	transfer	Tumor metastasis	0=No，1=Yes
14	targeted_therapy	Receiving targeted therapy	0=No，1=Yes
15	ALND	Axillary lymph node dissection	0=No，1=Yes
16	operation_type	Operation type	0=No，1=Breast conserving surgery，2=Radical operation
17	postoperative_chemotherapy	Postoperative chemotherapy	0=No，1=Yes
18	postoperative_radiotherapy	Postoperative radiotherapy	0=No，1=Yes
19	postoperative_targeting	Postoperative targeting	0=No，1=Yes

Supplementary Table 2 The baseline characteristics and clinical demographic data of the study population.

	training cohort (N=2540)	validation cohort (N=1101)	t/χ^2	P
**Age**				
Mean (SD)	54.3 (10.7)	53.8 (10.3)	1.44	0.15
Median [Min, Max]	53.0 [27.0, 92.0]	53.0 [27.0, 89.0]		
**Marital status**				
unmarried	2409 (94.8%)	1031 (93.6%)	2.122	0.145
married	131 (5.2%)	70 (6.4%)		
**Length of hospital stay**				
Mean (SD)	14.8 (5.57)	14.7 (5.73)	0.515	0.607
Median [Min, Max]	14.0 [1.00, 60.0]	14.0 [1.00, 56.0]		
**Histology grade**				
Grade1	99 (3.9%)	39 (3.5%)	8.45	0.015
Grade2	1782 (70.2%)	824 (74.8%)		
Grade3	659 (25.9%)	238 (21.6%)		
**Molecular subtyping**				
Luminal A	320 (12.6%)	153 (13.9%)	3.048	0.384
Luminal B	1539 (60.6%)	679 (61.7%)		
HER2 overexpressing	288 (11.3%)	109 (9.9%)		
Triple negative	393 (15.5%)	160 (14.5%)		
**Clinical stage**				
I	796 (31.3%)	372 (33.8%)	2.271	0.518
II	1165 (45.9%)	481 (43.7%)		
III	501 (19.7%)	215 (19.5%)		
IV	78 (3.1%)	33 (3.0%)		
**T-stage**				
T1	1189 (46.8%)	551 (50.0%)	4.196	0.241
T2	1151 (45.3%)	469 (42.6%)		
T3	118 (4.6%)	53 (4.8%)		
T4	82 (3.2%)	28 (2.5%)		
**N-stage**				
N0	1257 (49.5%)	557 (50.6%)	0.519	0.915
N1	798 (31.4%)	342 (31.1%)		
N2	273 (10.7%)	116 (10.5%)		
N3	212 (8.3%)	86 (7.8%)		
**M-stage**				
M0	2451 (96.5%)	1067 (96.9%)	0.407	0.524
M1	89 (3.5%)	34 (3.1%)		
**Tumor recurrence**				
No	2533 (99.7%)	1098 (99.7%)	<0.001	0.987
Yes	7 (0.3%)	3 (0.3%)		

**Tumor metastasis**				
No	1493 (58.8%)	650 (59.0%)	0.021	0.885
Yes	1047 (41.2%)	451 (41.0%)		
**Receiving targeted therapy**				
No	2105 (82.9%)	931 (84.6%)	1.575	0.21
Yes	435 (17.1%)	170 (15.4%)		
**Operation type**				
No	235 (9.3%)	122 (11.1%)		
Breast conserving surgery	434 (17.1%)	196 (17.8%)	3.529	0.171
Radical operation	1871 (73.7%)	783 (71.1%)		
**Axillary lymph node dissection**				
No	929 (36.6%)	428 (38.9%)	1.736	0.188
Yes	1611 (63.4%)	673 (61.1%)		
**Postoperative chemotherapy**				
No	441 (17.4%)	194(17.6%)	0.036	0.850
Yes	2099(82.6%)	907(82.4%)		
**Postoperative radiotherapy**				
No	1538 (60.6%)	661 (60.0%)	0.085	0.770
Yes	1002 (39.4%)	440 (40.0%)		
**Postoperative targeting**				
No	2203 (86.7%)	973 (88.4%)	1.859	0.173
Yes	337 (13.3%)	128 (11.6%)		
